# The temporal dynamics of sleep disturbance and psychopathology in psychosis: a digital sampling study

**DOI:** 10.1017/S0033291720004857

**Published:** 2022-10

**Authors:** Nicholas Meyer, Dan W. Joyce, Chris Karr, Maarten de Vos, Derk-Jan Dijk, Nicholas C. Jacobson, James H. MacCabe

**Affiliations:** 1 Department of Psychosis Studies, Institute of Psychology, Psychiatry and Neuroscience, King's College London, London, UK; 2 National Institute for Health Research (NIHR) Biomedical Research Centre at South London and Maudsley NHS Foundation Trust and King's College London, London, UK; 3 Department of Psychiatry, National Institute of Health Research, Oxford Health Biomedical Research Centre, Warneford Hospital, University of Oxford, Oxford, UK; 4 Audacious Technologies, Chicago, IL, USA; 5 Institute of Biomedical Engineering, University of Oxford, Oxford, UK; 6 ESAT, Department of Engineering & Department of Development and Regeneration, KU Leuven, Leuven, Belgium; 7 Sleep Research Centre, University of Surrey, Surrey, UK; 8 UK Dementia Research Institute, London, UK; 9 Center for Technology and Behavioral Health, Geisel School of Medicine, Dartmouth College, Lebanon, NH, USA

**Keywords:** Digital health, experience sampling, insomnia, mHealth, relapse prediction, schizophrenia

## Abstract

**Background:**

Sleep disruption is a common precursor to deterioration and relapse in people living with psychotic disorders. Understanding the temporal relationship between sleep and psychopathology is important for identifying and developing interventions which target key variables that contribute to relapse.

**Methods:**

We used a purpose-built digital platform to sample self-reported sleep and psychopathology variables over 1 year, in 36 individuals with schizophrenia. Once-daily measures of sleep duration and sleep quality, and fluctuations in psychopathology (positive and negative affect, cognition and psychotic symptoms) were captured. We examined the temporal relationship between these variables using the Differential Time-Varying Effect (DTVEM) hybrid exploratory-confirmatory model.

**Results:**

Poorer sleep quality and shorter sleep duration maximally predicted deterioration in psychosis symptoms over the subsequent 1–8 and 1–12 days, respectively. These relationships were also mediated by negative affect and cognitive symptoms. Psychopathology variables also predicted sleep quality, but not sleep duration, and the effect sizes were smaller and of shorter lag duration.

**Conclusions:**

Reduced sleep duration and poorer sleep quality anticipate the exacerbation of psychotic symptoms by approximately 1–2 weeks, and negative affect and cognitive symptoms mediate this relationship. We also observed a reciprocal relationship that was of shorter duration and smaller magnitude. Sleep disturbance may play a causal role in symptom exacerbation and relapse, and represents an important and tractable target for intervention. It warrants greater attention as an early warning sign of deterioration, and low-burden, user-friendly digital tools may play a role in its early detection.

## Introduction

Sleep disturbance is a common feature of the relapse prodrome of schizophrenia, reported in between 43% and 70% of patients (Birchwood et al., 1989; Heinrichs & Carpenter, 1985; Herz & Melville, 1980; Jorgensen, 1998), and appearing in the weeks prior to the onset of significant deterioration in mental state. These observations are clinically relevant, as they raise the possibility that dysregulated sleep is an important early warning sign of deterioration. If this were to be the case, sleep dysfunction may also play a causal role in psychotic deterioration and relapse, and present a tractable target for preventative interventions which avert progression to relapse. However, no studies have specifically examined the time-course of sleep disturbance in relation to the deterioration of psychosis.

Recent clinical studies have begun to examine the hypothesised association between sleep and psychopathology. In a 7-day experience sampling method (ESM) study of self-reported and actigraphic sleep variables on next-day symptoms in schizophrenia, Mulligan, Haddock, Emsley, Neil, and Kyle (2016) found that poorer sleep predicted greater next-day negative affect and psychosis symptoms, with negative affect mediating a proportion of this relationship. Similarly, in a 6-day ESM study in individuals on the psychosis-spectrum, Kasanova, Hajdúk, Thewissen, and Myin-Germeys (2020) reported a significant effect of poor sleep quality on paranoia the next morning, with negative affect fully mediating the relationship. Here, no evidence of an inverse relationship – greater paranoia on evening sleep quality – was found. A third study by Reeve, Nickless, Sheaves, and Freeman (2018) assessed subjective insomnia, psychosis and negative affect symptoms at monthly intervals over 3 months, in 29 patients with non-affective psychosis. Insomnia predicted later psychosis symptoms, and this relationship was again mediated by negative affect. This study also found a reciprocal effect between psychotic symptoms and insomnia, implying a bidirectional relationship between these variables.

Although canonical ESM offers high temporal resolution by intensively sampling mental state at multiple time-points throughout the day, the approach has some limitations. It is demanding of the participant, and lends itself to short periods of observation (Palmier-Claus et al., 2011). Analytic approaches have typically used multilevel models to examine short-term associations between variables from one day to the next, and are not optimised for exploring lag structures over longer timescales. Studies are required that are of sufficient duration to capture longitudinal fluctuations in sleep and psychopathology, while of adequate temporal resolution to sample variation over a range of timescales. Methods of analysis that allow the temporal structure between longitudinal variables to be examined are also necessary.

Designed collaboratively with service users, the *Sleepsight* platform (Meyer et al., 2018) capitalises on the ubiquity and usability of smartphone technologies to allow long-term sampling of self-reported sleep (sleep quality and duration) and psychopathology variables (positive affect, negative affect, cognition and psychosis symptom) in individuals living with psychosis, with minimal burden to the user. We captured these variables once a day over the course of a year, in 36 individuals with schizophrenia, collecting over 12 000 data-points.

**Fig. 1.**
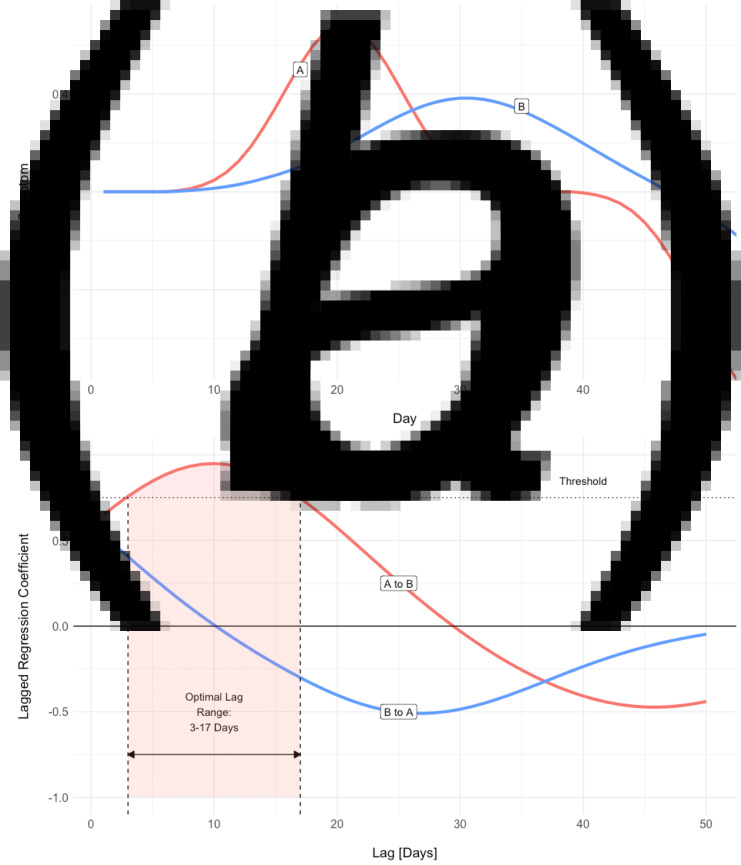
**(***a***)** Simulated time-series for one participant. An increase in the severity of symptom A appears to precede that of symptom B, and the temporal relationship is suggestive of A driving B. DTVEM looks for the same pattern across the entire time-series, for all participants. (*b*) Lagged regression between the two symptom time-series produces two coefficients, A to B and B to A. Here, symptom A maximally predicts symptom B over a threshold of statistical significance, with a lag of 3–17 days. However, symptom B does not significantly predict symptom A.

We employed an analytic tool developed specifically for investigating the temporal structure of intensive longitudinal data, the Differential Time-Varying Effect Model (DTVEM; Jacobson, Chow, & Newman, 2019). DTVEM examines the lag structure between one or more time-series, using a two-stage, exploratory–confirmatory model. This approach examines the lags at which one construct maximally predicts itself, or another construct, at a later time, and here allows the strength and timing of association between sleep and psychopathology to be studied (Fig. 1).

We predicted that shorter sleep duration and poorer sleep quality would be associated with greater intensity of psychotic, negative affect and cognitive symptoms. In the context of findings supporting a bidirectional relationship between sleep disturbances and psychopathology from the mood disorder literature (Talbot et al., 2012), we also expected to observe a reciprocal relationship between psychopathology and sleep, but predicted that this association would be of shorter duration and lower magnitude. In line with previous research, we also hypothesised that the relationship between sleep and psychosis symptoms would be mediated by negative affect but also cognitive symptoms, as evidenced by a weaker and less durable direct effect of sleep on psychosis when these variables are included as mediators.

## Methods

### Sleepsight app and platform

Developed specifically to optimise acceptability and feasibility in people with psychosis, the Sleepsight system consisted of an Android smartphone application and secure back-end infrastructure, for the real-time collection of self-reported sleep and psychopathology variables. Further details of the development and testing of the system (Biagianti, Hidalgo-Mazzei, & Meyer, 2017; Meyer et al., 2018) and source code for the application have been published elsewhere (Karr, 2017). Although Sleepsight also gathered continuous objective rest-activity variables from smartphone sensors and wearable devices, in the present paper, we focus uniquely on the self-reported, subjective variables.

**Fig. 2.**
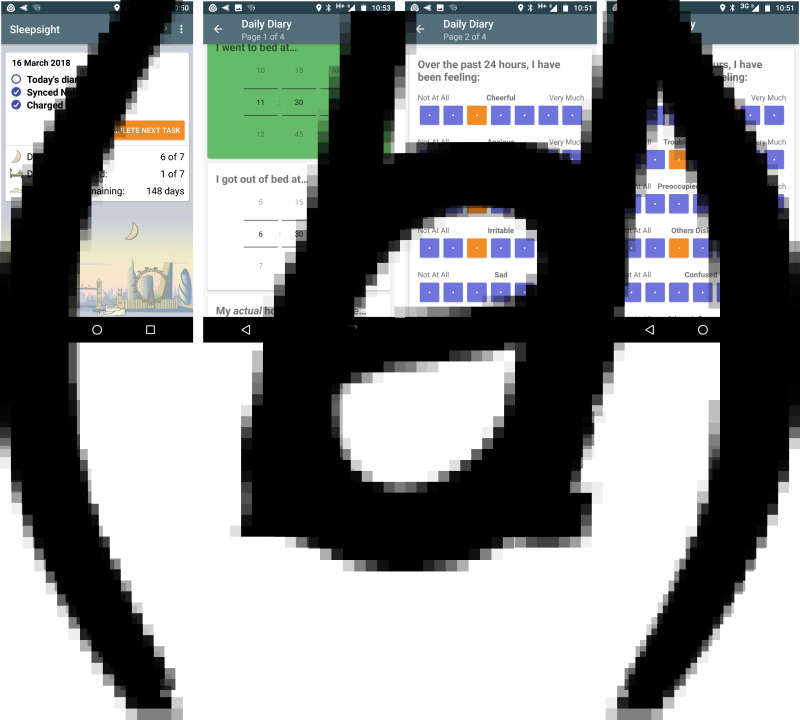
Screenshots of Sleepsight user-facing app: (*a*) splash page, (*b*) sleep diary, (*c*) mood symptoms, (*d*) psychosis symptoms. Note that only a section of screens (*b*–*d*) are visible.

A fixed sampling schedule prompted users to complete a brief once-daily sleep and symptom diary, between 11:00 and 14:00. This window was chosen to be early enough to minimise forgetting the previous night's sleep times, while also avoiding diurnal extremes of mood (Peeters, Berkhof, Delespaul, Rottenberg, & Nicolson, 2006). If not completed after the first notification, participants received two further prompts at 30 min intervals. The sleep diary (Fig. 2*b*) consisted of four items: time into bed, time out of bed, sleep duration and sleep quality, rated from 1 (‘very poor’) to 5 (‘very good’). All times were entered using a 12 h clock, with AM and PM specifiers, to the nearest 15 min. The symptom diary (Fig. 2*c*, *d*) consisted of items validated and widely used in previous ESM studies of psychosis (e.g. Myin-Germeys, Van Os, Schwartz, Stone, & Delespaul, 2001; Palmier-Claus *et al*. 2012). Rated on a seven-point Likert scale anchored at ‘not at all’ to ‘very much’, they consisted of seven mood items (‘over the past 24 h, I have been feeling: cheerful, anxious, relaxed, irritable, sad, in control, stressed’) and seven items relating to psychosis and cognition (‘over the past 24 h, I have been experiencing/feeling: suspicious, trouble concentrating, preoccupied by thoughts, others dislike me, confused, others influence my thoughts, unusual sights and sounds’). Positive and negative mood items were counterbalanced, to reduce skew and minimise stereotyped responses. The diary took approximately 1–3 min to complete, and all sections had to be completed in order for the app to allow submission. Participants received a motivational message on submitting a diary, but did not receive feedback based on their entries.

### Participants and study procedures

Adults aged 18–65, living in the community with a diagnosis of schizophrenia or schizoaffective disorder, and under the care of secondary care mental health services were invited to take part. Participants were required to be able to provide informed consent and interact comfortably with the smartphone interface, but were otherwise not excluded on the severity of their psychosis symptoms or medication status.

On study entry, the Positive and Negative Syndrome Scale (PANSS), Pittsburgh Sleep Quality Index (PSQI; Buysse, Reynolds, Monk, Berman, & Kupfer, 1989) and Insomnia Severity Index (ISI; Bastien, Vallières, & Morin, 2001) were administered. Participants without their own device were provided with an Android smartphone, which they were asked to use as their sole device throughout the study period. For eligible participants already in possession of an Android device, the Sleepsight app was installed on their own device. Therefore, the risk of non-response by having multiple devices was minimised, by ensuring all participants only made use of one device. All participants received equal compensation for their time, regardless of device ownership status, and received a face-to-face training session on enrolment.

Participants were asked to respond to the once-daily smartphone notification by completing the smartphone diary, and use their mobile devices as normal, for 12 months. Participants received a weekly motivational SMS message, and were contacted if they were having technical difficulties, or if the engagement was low. Otherwise, no change was made to the delivery of psychiatric care. Screening for eligibility and all clinical assessments were undertaken by an experienced psychiatrist (NM), and the study received ethical approval from the local Research Ethics Committee.

### Data analysis

**Fig. 3.**
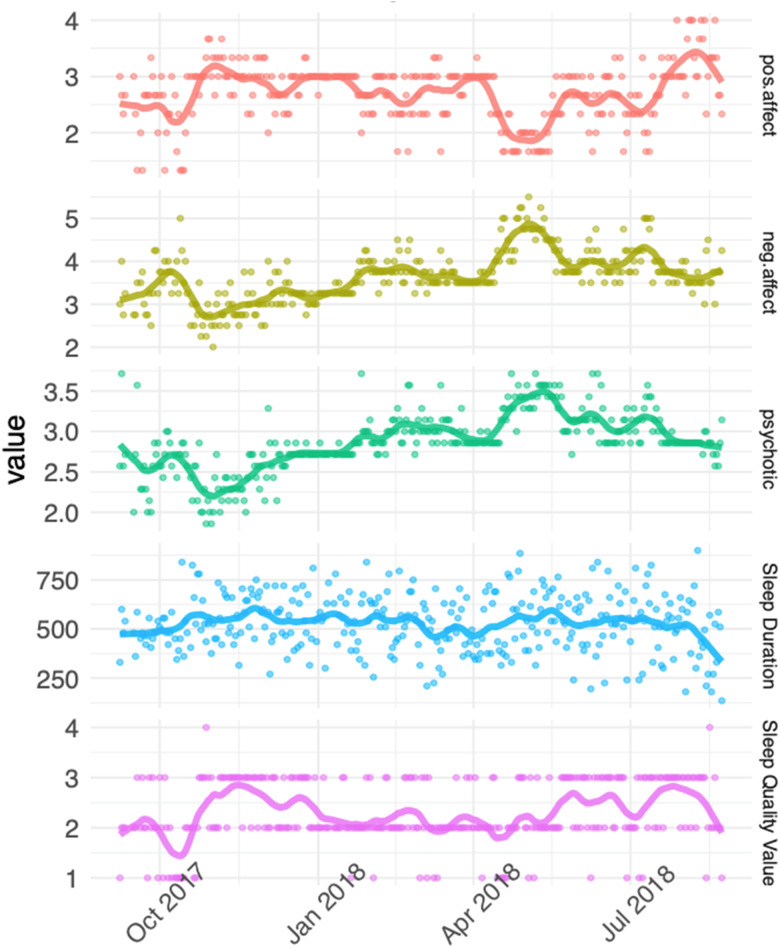
Example responses over 12 months from one participant, fitted with a loess smoother with degree 1 polynomial. Higher scores indicate better sleep quality and positive affect, longer sleep duration, and worse negative affect and psychosis symptoms.

Data were organised with the unit of time as one day. Four summary psychopathology variables were computed from diary items: positive affect (mean of ‘cheerful, ‘relaxed’ and ‘in control’); negative affect (mean of ‘anxious’, ‘irritable’, ‘sad’, ‘stressed’), cognitive symptoms (mean of ‘trouble concentrating’, ‘confused’) and psychosis symptoms (mean of ‘suspicious’, ‘preoccupied by thoughts’, ‘others dislike me’, ‘others influence my thoughts’ and ‘unusual sights and sounds’). In order to examine associations between sleep and individual psychotic symptoms in more detail, two additional symptoms were included in a secondary analysis: hallucinations (i.e. the ‘unusual sights and sounds’ item) and paranoia (mean of ‘suspicious’ and ‘others dislike me’ items). Duplicate entries within a 24 h period were removed, and summary variables visualised (Fig. 3). Participants whose data showed minimal variation, suggestive of stereotyped responses, were excluded, and the rate of diary completion over the 12-month period was calculated.

The predictive lag structure between longitudinal variables was then estimated using *DTVEM* (Jacobson et al., 2019), which estimates the lags at which one variable predicts another, over a threshold of statistical significance from a multi-stage modelling procedure (Fig. 1). In the exploratory stage, *DTVEM* uses generalised additive mixed models [GAMMs, as part of R package *mgcv* (Wood, 2011)] to identify lags that have substantial effects on the outcome variable, thereby narrowing down the search space and improving the computational efficiency of the confirmatory step. Next, *DTVEM* passes these to a vector autoregressive of order *p* [VAR(*p*)] model [using the *OpenMx* (Neale et al., 2016) R package], which uses a state-space modelling framework to confirm when one variable maximally predicts another. *DTVEM* cycles iteratively between stages until an optimal solution is found. We only report lag relationships between variables which DTVEM has identified as statistically significant, and also report the combined total effect size of all significant lags between two variables, rather than the effect sizes of each individual lag. *DTVEM* has been shown to be robust to missing data. This two-stage approach has been shown to be conservative in dramatically reducing type I error, while retaining high power.

In the bivariate, unmediated analyses, a single predictor (sleep quality or duration) and outcome (psychopathology variable) were modelled, to establish the total unadjusted lag relationship (i.e. the *total effect* between the two variables). Next, the variables were reversed, to examine the reciprocal lag relationship between psychopathology and sleep. An auto-regression model was also fitted to examine whether sleep variables predicted themselves over time.

The mediation analyses followed the approach of Baron and Kenny (1986) and used the joint significance test, which has been shown to perform better than many of the alternative methods (Leth-Steensen & Gallitto, 2016). First, the putative mediator variable (negative affect or cognitive symptoms) was fitted to the predictor (sleep variable): the absence of a predictive relationship would suggest that the intermediate variable could not act as a mediator. Next, the mediator and predictor were entered together in the same model, with psychosis symptoms as the outcome variable. This allows estimation of the *direct effect* between predictor and outcome in the presence of the mediator, and the *indirect effect* between predictor and outcome *via* the mediator. A reduction in the size of the *direct effect* in comparison with the *total effect* would support the presence of the mediator, and favour the mediated model. Also, model fit for the unmediated and mediated analyses was compared using the Bayesian Information Criteria (BIC), which penalises for the complexity of the model and sample size. A lower BIC indicates a closer fit of model to the data. Finally, as negative affect and cognitive symptoms are hypothesised to have a reciprocal relationship on sleep, an oppositional model was examined, where the above steps were repeated with the sleep variable as the mediator and negative affect/cognitive symptoms as the predictor. The effect sizes and model fit of both models were compared, and the model which provided the most parsimonious explanation for the data was favoured.

For all analyses, a lag window of 1–20 days was chosen. Given that each analysis is computationally intensive, this strikes a balance between reducing analysis time, while allowing a realistic window of time within which early signs of relapse have been reported in previous studies (e.g. Birchwood et al., 1989; Heinrichs & Carpenter, 1985; Herz & Melville, 1980; Jorgensen, 1998). All variables were standardised to obtain person-centred *z*-scores, and the number of *k* selection points was set at 19 (i.e. lag window −1). Analyses were undertaken in R (R Core Team, 2020), using the Rosalind High Performance Computing Infrastructure, King's College London.

## Results

### Demographic and clinical characteristics

Characteristics of the 36 individuals who took part in the study are presented in Table 1. Six participants were within the first 5 years of their first episode of psychosis and therefore under the care of early intervention mental health services, while the remaining participants had a more established illness. Thirty-five out of 36 participants were prescribed regular antipsychotic medication, of which nine (25%) were taking clozapine for treatment-resistant psychosis, nine (25%) were prescribed more than one antipsychotic and seven (19%) were receiving depot medication. The ISI score for 11 (31%) participants was within the range of clinically significant insomnia (score of 15 or above), and the PSQI score indicated poor sleep quality (score of 5 or greater) for 26 (72%) participants.

**Table 1.** Demographic and clinical characteristics on study entry (*n* = 36), and summary statistics of self-reported sleep variables (*n* = 33)

	Mean	s.d.	Min	Max	Range
Sex					
Male *n* (%)	24 (67)				
Female *n* (%)	12 (33)				
Age/years	40.7	9.7	20.0	59.0	39.0
Duration of illness/years	14.4	8.4	0.8	30.0	29.2
PANSS total score	73.1	16.6	44.0	115.0	71.0
Positive subscale	16.8	4.8	7.0	25.0	18.0
Negative subscale	19.1	7.1	7.0	35.0	28.0
General subscale	37.2	8.5	21.0	57.0	36.0
BDI-II	18.3	11.4	1.0	49.0	48.0
PSQI total score	7.5	4.0	1.0	15.0	14.0
ISI	12.7	6.9	0.0	25.0	25.0
ESS	6.0	5.0	0.0	23.0	23.0
STOP-BANG	2.4	1.6	0.0	6.0	6.0
Self-reported sleep variables					
Sleep duration/min	489.8	160.7	0	1425	1425
Sleep quality	3.24	1.06	1	6	6

The mean (s.d.) duration of participation was 323 (88) days, number of submitted diaries per participant was 246 (136), and the overall response rate was 69%. Data from two participants who were in the study for <1 month, and one participant whose responses showed no variability, were excluded; therefore, data from 33 participants were entered into the final analysis.

### Unmediated associations between sleep and psychopathology

Sleep quality negatively predicted psychosis symptoms over lags of 1–8 days (i.e. better sleep quality was associated with lower psychosis symptoms over an 8-day horizon, and *vice versa*). It also negatively predicted negative affect over lags of 1–8 and 10–11 days, and cognitive symptoms over lags of 1–8 days. Conversely, sleep quality positively predicted positive affect over 1–5 days (i.e. better sleep quality was associated with greater positive affect, over a 5-day window). Effect sizes were in the medium range.

Sleep duration negatively predicted psychosis symptoms over lags of 1–12 days, negative affect over lags of 1–8 days, and cognitive symptoms over lags of 1, 3–12 and 14–15 days. No association between sleep duration and positive affect was found (Table 2, top).

**Table 2.** Self-reported sleep variables: lag relationships between single predictor–outcome pairs

Predictor variable	Outcome variable	Polarity and duration of lag/days	Effect size^a^
Sleep variables predicting psychopathology			
Sleep quality	Psychosis symptoms	−(1–8)	−0.567
Sleep quality	Paranoia	−(1:5)	−0.502
Sleep quality	Hallucinations	−(1:5)	−0.329
Sleep quality	Negative affect	−(1–8, 10–11)	−0.590
Sleep quality	Positive affect	+(1–5)	0.509
Sleep quality	Cognitive symptoms	−(1–8)	−0.464
Sleep duration	Psychosis symptoms	−(1–12)	−0.501
Sleep duration	Paranoia	n.s.	–
Sleep duration	Hallucinations	−(2–3, 5–9)	−0.332
Sleep duration	Negative affect	−(1–8)	−0.446
Sleep duration	Positive affect	n.s.	–
Sleep duration	Cognitive symptoms	−(1, 3–12, 14–15)	−0.511
Psychopathology predicting sleep variables			
Psychosis symptoms	Sleep quality	−(1–3)	−0.203
Paranoia	Sleep quality	−(1–2, 3, 6)	−0.221
Hallucinations	Sleep quality	n.s.	–
Negative affect	Sleep quality	−(1–3, 5–6)	−0.301
Positive affect	Sleep quality	+(1–3, 6–7)	0.359
Cognitive symptoms	Sleep quality	−(1–2, 4–5)	−0.220
Psychosis symptoms	Sleep duration	n.s.	–
Paranoia	Sleep duration	n.s.	–
Hallucinations	Sleep duration	n.s.	–
Negative affect	Sleep duration	n.s.	–
Positive affect	Sleep duration	n.s.	–
Cognitive symptoms	Sleep duration	n.s.	–
Auto-correlation of sleep and psychopathology variables			
Sleep quality	Sleep quality	+(1–5)	0.625
Sleep duration	Sleep duration	+(1–4, 6–8, 10, 14, 19)	0.609
Psychosis symptoms	Psychosis symptoms	+(1–6)	0.865
Negative affect	Negative affect	+(1–4)	0.783
Positive affect	Positive affect	+(1–3)	0.707
Cognitive symptoms	Cognitive symptoms	+(1–5)	0.794

n.s., no significant lag identified.

a Sum of *β* coefficients over all lags.

### Reciprocal associations between psychopathology and sleep variables

Examining the opposite causal direction, statistically significant negative associations of smaller effect size were found between psychosis symptoms and sleep quality (over lags of 1–3 days), negative affect and sleep quality (over lags of 1–3 and 5–6 days), and cognitive symptoms and sleep quality (with lags of 1–2 and 4–5 days). Positive affect predicted better sleep quality over lags of 1–3 and 6–7 days.

No associations were found between psychosis symptoms and sleep duration, positive or negative affect and sleep duration, or cognitive symptoms and sleep duration (Table 2, middle).

### Unmediated associations between sleep and individual psychotic symptoms

Sleep quality had a negative relationship with both paranoia and hallucinations, over a lag of 1–5 days, in the low–medium effect size range. In the reciprocal direction, only paranoia, and not hallucinations, predicted sleep quality.

Sleep duration predicted hallucinations, but not paranoia, with lags of 2–3 and 5–9 days. No reciprocal association between individual psychotic symptoms and sleep duration was found.

### Auto-regression analyses in sleep and psychopathology variables

Sleep quality predicted itself over lags of 1–5 days (i.e. better sleep quality was associated with better sleep quality over the next five nights), and sleep duration predicted itself over lags of 1–4, 6–8, 10, 14 and 19 days. Negative affect, positive affect, cognitive and psychosis symptoms predicted themselves with lags of 1–4, 1–3, 1–5 and 1–6 days, respectively, with effect sizes in the medium–large range (Table 2, bottom).

### Mediated associations between sleep and psychopathology

Results of mediation analyses are set out in Table 3 and explained in greater detail in online Supplementary data and Figs S1–S4. In all cases, models where negative affect and cognitive symptoms mediated the relationship between sleep quality/sleep duration and psychosis symptoms were favoured over unmediated relationships. Negative affect was a full mediator of the effect between sleep duration and psychosis symptoms, and a partial mediator of the effect between sleep quality and psychosis symptoms. Cognitive symptoms were a partial mediator of the effect between sleep quality and sleep duration, and psychosis symptoms.

**Table 3.** Mediated lag relationships and effect sizes

Model	Predictor	Mediator	Outcome	Total effect, lag	Direct effect, lag	Indirect effect, (path A), lag	Indirect effect, (path B), lag	BIC, unmediated model	BIC, mediated model	Preferred model
1a	Sleep duration	Negative affect	Psychosis symptoms	−0.501 −(1–12)	n.s. (–)	−0.446 −(1–8)	0.706 +(1–3)	190 964	61 874	Fully mediated
1b	Negative affect	Sleep duration	Psychosis symptoms	0.704 +(1–3)	0.706 +(1–3)	n.s. (–)	n.s. (–)	40 454	61 874	Unmediated
2a	Sleep quality	Negative affect	Psychosis symptoms	−0.567 −(1–8)	−0.072 −(2–4)	−0.590 −(1–8, 10–11)	0.688 +(1–3)	190 531	61.691	Partially mediated
2b	Negative affect	Sleep quality	Psychosis symptoms	0.704 +(1–3)	0.688 +(1–3)	−0.301 −(1–3, 5–6)	−0.072 −(2–4)	40 454	61 691	Unmediated
3a	Sleep duration	Cognitive symptoms	Psychosis symptoms	−0.501 −(1–12)	−0.054 −(3–6)	−0.511 −(1, 3–12, 14–15)	0.613 +(1–2)	190 964	62 147	Partially mediated
3b	Cognitive symptoms	Sleep duration	Psychosis symptoms	0.875 +(1–7)	0.613 +(1–2)	n.s. (–)	−0.054 −(3–6)	40 065	62 147	Unmediated
4a	Sleep quality	Cognitive symptoms	Psychosis symptoms	−0.567 −(1–8)	−0.115 −(1–3)	−0.464 −(1–8)	0.896 +(1–10)	190 531	61 233	Partially mediated
4b	Cognitive symptoms	Sleep quality	Psychosis symptoms	0.875 +(1–7)	0.896 +(1–10)	−0.220 −(1–3, 5–6)	−0.115 −(1–3)	40 065	61 233	Unmediated

n.s., no significant lag identified.

Total, direct and indirect effects are calculated from the sum of *β* coefficients over all lags. Refer to online Supplementary Figs S1–S4 for path diagrams.

Oppositional models where sleep variables mediated the relationship between negative affect/cognitive symptoms and psychosis symptoms either showed no evidence of an indirect pathway or showed poorer model fit, than the non-mediated total effect, arguing against an indirect relationship mediated by sleep duration or sleep quality.

## Discussion

### Main findings

Sleep disruption has traditionally been considered a consequence of psychosis, arising as the product of mental state disturbance. The possibility that it may play a causal role in the emergence of psychotic symptoms has received support only more recently, through studies in clinical (Kasanova et al., 2020; Mulligan et al., 2016; Reeve et al., 2018; Waters et al., 2011) and non-clinical populations (Freeman et al., 2017; Hennig, Schlier, & Lincoln, 2019; Reeve, Emsley, Sheaves, & Freeman, 2018; Scott, Rowse, & Webb, 2017). Using a purpose-built digital platform and novel analytic approach, our findings extend those of recent studies, by determining the temporal relationship over which sleep and psychopathology variables influence and sustain one another.

First, these results support the hypothesis that poorer sleep quality and sleep duration are an antecedent to worsening psychotic symptoms, occurring up to 8 and 12 days prior to the deterioration in mental state, respectively. Poorer sleep quality and duration also anticipate worsening in negative affect and cognitive symptoms, from 1 to 15 days before the deterioration in mood and cognition. Similarly, better sleep quality (but not sleep duration) predicted greater positive affect, but with a shorter lag duration of up to 5 days.

Second, we also found evidence of a reciprocal relationship, where greater psychopathology predicted poorer sleep quality (but interestingly, not sleep duration), and greater positive affect predicted better sleep quality. As advanced in previous reports (Harvey, Murray, Chandler, & Soehner, 2011; Talbot et al., 2012), this supports the concept of a bidirectional, mutually reinforcing association between sleep quality and psychopathology. Importantly, however, with the exception of positive affect, where the reciprocal association was of longer duration, the lags of reciprocal associations for all other variables were only between 1 and 6 days and of smaller effect size, suggesting that sleep has a more durable and pronounced effect on psychopathology than the opposite direction of the association. This finding is compatible with other studies, which found unidirectional (Kasanova et al., 2020) or only partially reciprocal (Hennig et al., 2019; Reeve et al., 2018) relationships between psychopathology and sleep variables.

It is notable that bidirectional associations were found for sleep quality, but not duration. This provides support to the argument that changes in the quantity and continuity of sleep drive the development of mental symptoms, but are in themselves relatively unaffected by mental state fluctuations. One factor that may contribute to this finding is the use of sedative antipsychotic medications in all but one of the participants, which have been shown to increase sleep duration and reduce sleep latency (Cohrs, 2008; Meyer et al., 2020), thereby dampening the impact of changes in psychopathology on sleep duration. Another explanation for this finding is that the subjective evaluation of sleep duration is less affected by past mental state, whereas sleep quality is more contingent on an individual's sense of well-being. That is, sleep quality is more closely ‘tied’ to psychopathology than is sleep duration.

Third, the mediation analyses provided stronger support for models where negative affect and cognitive symptoms mediated the relationship between sleep quality/sleep duration and psychosis symptoms, than unmediated and oppositional models, where sleep variables were the mediator. This replicates previous findings (Kasanova et al., 2020; Reeve et al., 2018; Scott et al., 2017), and reinforces the view that affective and cognitive processes lie on the pathway to psychotic symptoms. In exploring the relationship with individual psychotic symptoms, a bidirectional relationship between sleep quality and paranoia, and unidirectional association between sleep quality and hallucinations, was found. Also, changes in sleep duration influenced hallucinations, but not paranoia. This also accords with previous findings, and supports the hypothesis that the pathway between sleep disturbances and paranoia may differ from those linking sleep disturbances and hallucinations (Hennig et al., 2019; Reeve et al., 2018; Scott et al., 2017).

### Clinical implications and mechanisms

As conceived in the criteria of Bradford Hill (Bradford, 1965), temporality and strength of association are key tenets for inferring a causal relationship between a predictor and outcome. In this study, the longer lag duration and larger effect size when sleep variables are predictors, rather than outcome variables, suggest that sleep disturbance emerges earlier in the pathway to deterioration of psychosis, and supports a causal role for sleep disturbance in the development of psychopathology. A corollary of this is that subjective sleep disturbances may serve as a clinically useful early warning sign of impending deterioration, and allow the implementation of timely, preventative interventions. Our findings suggest that a reduction in sleep duration precedes psychotic deterioration by up to 12 days, suggesting that this is a particularly important symptom in this regard. A further implication is that psychological and pharmacological interventions which specifically target sleep dysfunction may diminish psychopathology and risk of relapse – a strategy which has gained recent experimental support (Freeman et al., 2017).

Another essential criterion for inferring causality is the presence of plausible mechanisms that link predictor and outcome variables. Experimental sleep deprivation in healthy volunteers induces depressed mood and anxiety, executive function and working memory impairment, perceptual distortions and hallucinations, and paranoia, with particularly strong associations with mood and cognitive variables (Kahn-Greene, Killgore, Kamimori, Balkin, & Killgore, 2007; Petrovsky et al., 2014; Reeve et al., 2018; Waters, Chiu, Atkinson, & Blom, 2018). These effects may be mediated by multiple biological mechanisms, including increased dopamine release and sensitivity (Yates, 2016), impaired sleep-dependent plasticity (Pocivavsek & Rowland, 2018) and disrupted synaptic homeostasis (Cirelli & Tononi, 2015). These findings also align with theoretical frameworks which implicate negative affect, worry and rumination (Hartley, Haddock, Vasconcelos e Sa, Emsley, & Barrowclough, 2014), and cognitive impairment (Garety *et al*., 2013) in driving psychotic symptoms – constructs which were also identified as mediators in this study. There is also growing interest in the role of immune dysregulation in the pathoaetiology of psychosis (Pillinger et al., 2018), which converges with evidence suggesting that impaired sleep plays a causal role in the development of metabolic and cardiovascular disease (Clark et al., 2016), mediated by inflammatory mechanisms (Irwin, Olmstead, & Carroll, 2016; Mullington, Simpson, Meier-Ewert, & Haack, 2010).

### Strengths and limitations

A key feature of this study is its use of novel approaches for sampling and analysing intensive longitudinal data that goes beyond single-day lags, and allows the evaluation of longer lag structures between dynamic variables over ecologically valid timescales. There were high levels of engagement with the technology for such a study, yielding a high response rate and an unparalleled dataset.

However, it is important to note that *DTVEM* operates by examining relationships between multiple concurrent processes in relative time, rather than absolute time, and assumes that the relationship between covariates does not change as a function of study time. Related to this, deterioration in sleep and mental state may occur contemporaneously; however, it is possible the current study design lacks the sensitivity to detect more subtle, early shifts in psychopathology. Also, *DTVEM* remains a novel method, which requires further validation and replication. The study was promoted as one which examined sleep, meaning that individuals with sleep disorders may have been over-represented in our sample. However, rates of insomnia in this study were comparable to those published elsewhere (Palmese et al., 2011; Xiang et al., 2009). We cannot exclude the possibility that the response rate to the daily diary varies with symptom severity at baseline, and also with longitudinal changes in mental state, leading to non-response biases. Finally, given the limitations inherent to self-report measures, including noise and non-response, further examination of the nature of sleep disturbance using objective measures and a better understanding of how these measures relate to sleep quality (Della Monica, Johnsen, Atzori, Groeger, & Dijk, 2018) are warranted.

## Conclusions

Deterioration in sleep quality and sleep duration predicts worsening of psychosis symptoms from up to 8 and 12 days in advance, respectively, and is mediated by negative affect and cognitive symptoms. Psychopathology variables have a reciprocal predictive effect on sleep quality, supporting a bidirectional relationship, but is generally weaker and of shorter duration.

These findings support the conclusion that sleep disturbance plays a causal role in symptom exacerbation and relapse in psychosis, and suggests it warrants greater attention as an early warning sign of deterioration.

## Supplementary material

For supplementary material accompanying this paper visit https://doi.org/10.1017/S0033291720004857. click here to view supplementary material
