# Supplementary material for: The temporal dynamics of sleep disturbance and psychopathology in psychosis: a digital sampling study
Source: Psychol Med. 2021 Jan 12;52(13):2741–50. doi: 10.1017/S0033291720004857 (PMC9647520; doi:10.1017/S0033291720004857)
Supplement: Supplementary file 1 [file S0033291720004857sup001.pdf]

SUPPLEMENTARY MATERIAL TO:

TEMPORAL DYNAMICS OF SLEEP DISTURBANCE AND PSYCHOPATHOLOGY IN PSYCHOSIS: A DIGITAL SAMPLING STUDY

1. Supplementary Results

In model 1a (fig 1) both an unmediated and fully mediated pathway between sleep duration and psychosis symptoms are possible. However, a lower Bayesian information criterion (BIC) in the mediated model suggests that this model provides a better fit to the data. In oppositional model 1b, a mediated pathway is not possible as indirect pathway A' is not significant. Therefore, an unmediated pathway between negative affect and psychosis is a more parsimonious explanation.

In model 2a (fig 2) both unmediated and partially mediated mediated pathways between sleep quality and psychosis symptoms are possible. The lower BIC in the mediated model favours this pathway. In oppositional model 2b, both an unmediated and mediated model are possible, however the lower BIC in the unmediated model favours this direct pathway.

In model 3a (fig 3), both an unmediated and partially mediated pathway between sleep duration and psychosis symptoms are feasible, however the lower BIC in the mediated pathway favours this pathway over the unmediated route. In oppositional model 3b, a mediated pathway is not possible as indirect pathway A'_2 between cognitive symptoms and sleep duration is not significant, favouring an unmediated pathway between cognitive symptoms and psychosis symptoms.

In model 4a (fig 4) both an unmediated and partially mediated pathway are possible, however the lower BIC in the mediated model suggests this is a better fit to the data. In the oppositional model 4b, both an unmediated and mediated pathway are possible, however the lower BIC favours the former pathway.

2. Supplementary Figures

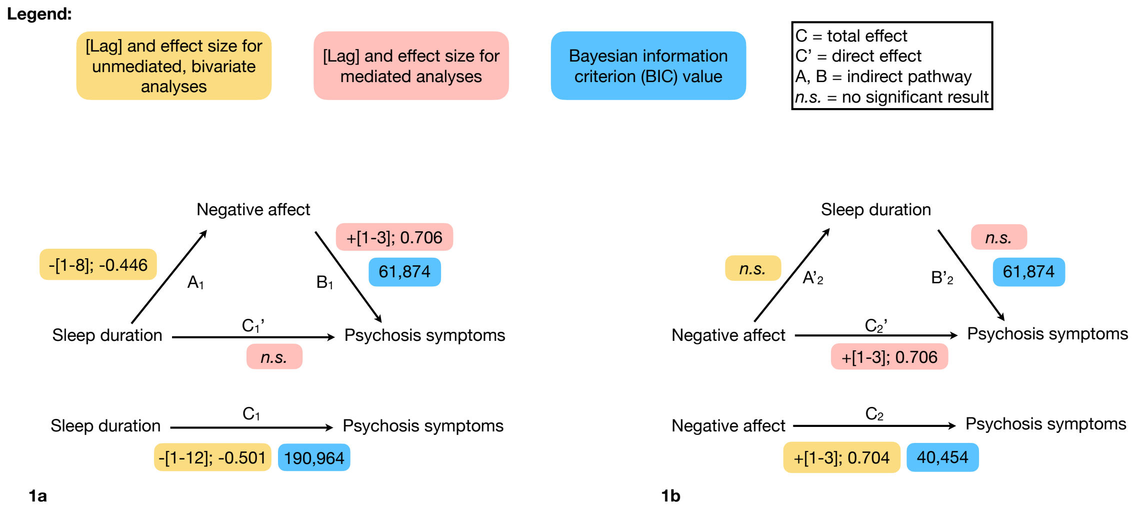

Figure 1: Model 1a: sleep duration predicting psychosis symptoms, moderated by negative affect. Model 1b: negative affect predicting psychosis symptoms, moderated by sleep duration.

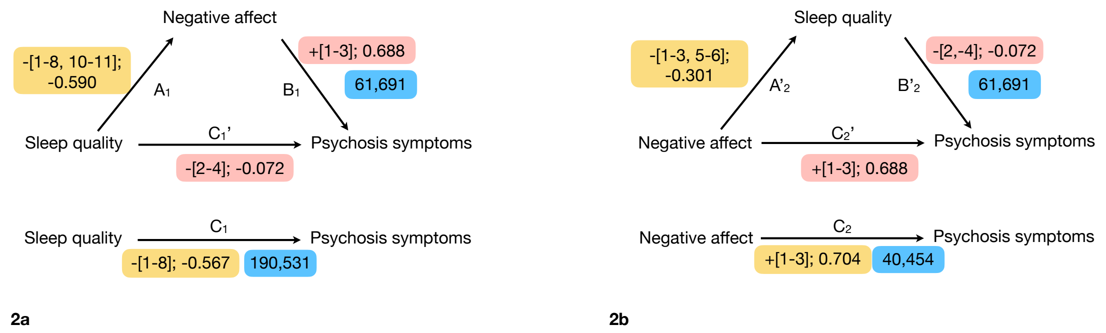

Figure 2: Model 2a: sleep quality predicting psychosis symptoms, moderated by negative affect. Model 2b: negative affect predicting psychosis symptoms, moderated by sleep quality.

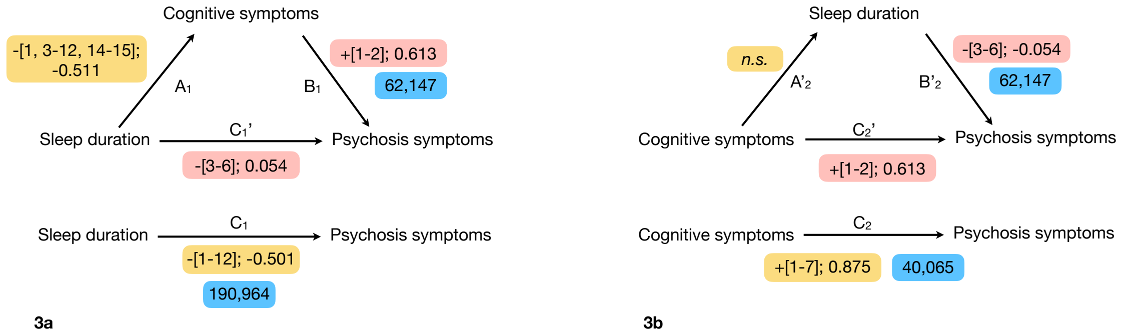

Figure 3: Model 3a: sleep duration predicting psychosis symptoms, moderated by cognitive symptoms. Model 3b: cognitive symptoms predicting psychosis symptoms, moderated by sleep duration.

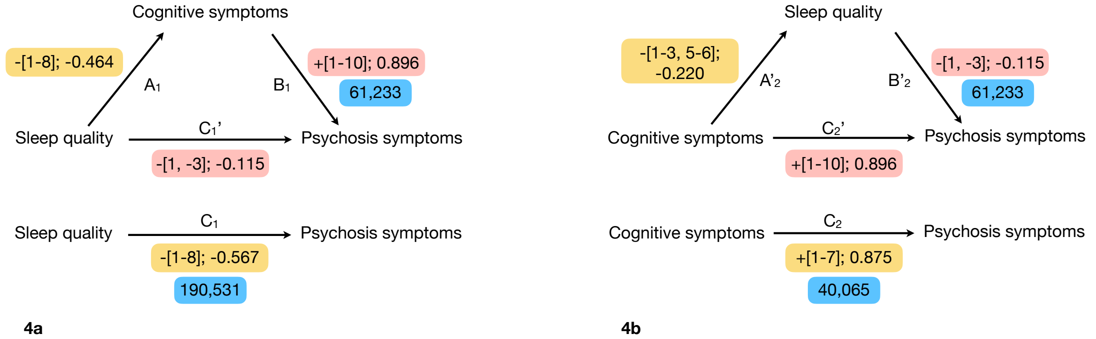

Figure 4: Model 4a: sleep quality predicting psychosis symptoms, moderated by cognitive symptoms. Model 4b: cognitive symptoms predicting psychosis symptoms, moderated by sleep quality.
